# Mapping Assessments Instruments for Headache Disorders against the ICF Biopsychosocial Model of Health and Disability

**DOI:** 10.3390/ijerph18010246

**Published:** 2020-12-31

**Authors:** Domenico D’Amico, Stewart J. Tepper, Erika Guastafierro, Claudia Toppo, Matilde Leonardi, Licia Grazzi, Paolo Martelletti, Alberto Raggi

**Affiliations:** 1 UOC Neuroalgologia, Fondazione IRCCS Istituto Neurologico Carlo Besta, 20133 Milan, Italy; licia.grazzi@istituto-besta.it; 2 Department of Neurology, Geisel School of Medicine at Dartmouth, Hanover, NH 03755, USA; stewart.j.tepper@dartmouth.edu; 3 UOC Neurologia, Salute Pubblica, Disabilità, Fondazione IRCCS Istituto Neurologico Carlo Besta, 20133 Milan, Italy; erika.guastafierro@istituto-besta.it (E.G.); claudia.toppo@istituto-besta.it (C.T.); matilde.leonardi@istituto-besta.it (M.L.); alberto.raggi@istituto-besta.it (A.R.); 4 Department of Clinical and Molecular Medicine, Sapienza University, 00185 Rome, Italy; paolo.martelletti@uniroma1.it

**Keywords:** migraine, tension-type headache, cluster headache, disability, quality of life, MIDAS, HIT-6, MSQ, HEADWORK

## Abstract

Headache disorders have a strong impact on sufferers’ lives. However, the “content” of assessment instruments addressing concepts, such as disability and quality of life (QoL), has not comprehensively been addressed. We searched SCOPUS for research papers in which outcome measures were used in adult populations of patients with migraine, tension-type headache (TTH), and cluster headache (CH). The content of single instruments was then mapped against the International Classification of Functioning, Disability, and Health. A total of 150 papers and 26 instruments were included: 15 addressed disability or impact, two addressed work-related difficulties, and nine addressed QoL. Few instruments were commonly used across the conditions and covered domains of functioning were impact on daily life activities, homework, school, and work-related tasks, leisure time, informal and family relations, pain, emotional difficulties, energy level, and impulse control. Most of the research is based on instruments that were developed for migraine, which is critical for CH, and the impact of headache disorders on work-related activities is poorly acknowledged. Further research is needed to expand the scope of headaches impact on daily life activities, and on environmental factors relevant to headache disorders to raise knowledge on the less represented areas, e.g., TTH impact.

## 1. Introduction

Headache disorders are among the most prevalent, disabling, and burdensome conditions worldwide. As shown in the 2017 report from the Global Burden of Disease (GBD) study, headache disorders were stably ranked second throughout the 1990–2017 period in terms of prevalence and in terms of years lived with a disability (YLDs), both among females and males [1]: they represent 42% of all-causes in terms of prevalence and 6% of all-cause YLDs. Headache disorders account for 98% of prevalence of neurological disorders—mostly due to tension-type headache (TTH), which accounts for 76% of headache disorders prevalence—and for 74% of the YLDs attributed to neurological disorders—mostly due to migraine, which accounts for 87% of disability due to headache disorders. In terms of Disability-Adjusted Life Years (DALYs), a measure of burden which accounts for both disability and mortality, headache disorders were in the 8th ranked position for females and in the 18th ranked position for males in 2017 (moving from the 12th and 24th ranked position of 1990, respectively) [2]. The reason for this is that there is no mortality directly associated with headache disorders, and therefore the burden of headaches is entirely a matter of disability and impact on patients’ daily life.

Such a concept has been explored in some literature reviews, which showed that the impact of headache disorders is mostly connected with issues such as emotional problems, reduced vitality and fatigue, pain, reduced physical and mental health, poor social functioning, and increased global disability, speaking, watching and reading, focusing attention, problem solving and handling stress, driving, family relations, and difficulties at work [3,4,5]. Published results are mostly connected to migraine, and more specifically to chronic migraine (CM), and obtained through the use of few assessment instruments, namely the Migraine Disability Assessment scale (MIDAS) [6], the six-Item Headache Impact Test (HIT-6) [7], and the Migraine-Specific Quality of Life Questionnaire (MSQ) [8]. There is a lack of information about TTH and poor information about cluster headache (CH) [9], a condition in which most of research was designed using again the HIT-6, MIDAS, and the MSQ. The large reliance on these assessments poses some problems, as MIDAS and MSQ were designed for migraine, and not for TTH or CH. The use of MIDAS is particularly critical in CH, where the number of attacks, their intensity, duration, and time to relief are clinical outcomes of higher interest [10,11], rather the number of days with headache in a given period, which is the focus of MIDAS. Finally, the use of MIDAS is critical in CM too, given the long recall period (90 days) and the large amount of days with migraine headache. There is a likelihood that patients with CM approximate their response to a “round” number—such as multiples of 10 or 5—higher compared to what patients with an episodic form do [12], thus posing reliability concerns on MIDAS use in CM.

Notwithstanding the criticism on MIDAS use in some specific populations, it is a fact that it is widely used in headache research, together with the HIT-6 and the MSQ. However, these patient reported outcomes (PROs) and other assessments used to address headache disorders’ disability and impact, have been generally limited to migraine only. The International Classification of Functioning, Disability, and Health (ICF) [13] is the best taxonomy to address the need for more widespread and applicable disability PROs, as it enables identification of what areas of human functioning are represented. The ICF classifies human functioning and disability in terms of impairments in body functions and body structures (BF and BS), in terms of limitations in activities and participation (A&P) and also includes a comprehensive taxonomy of environmental factors (EF) that may act as barriers which reduce functioning and increase disability, or facilitators that, on the contrary, promote functioning and reduce disability.

An ICF-based approach to the identification of the most relevant areas of functioning has been used in different conditions, including migraine, through a set of systematic literature reviews in which the information from a given assessment used to describe functioning in conditions such as depression, schizophrenia, alcohol dependence, epilepsy, and migraine have been cross-linked to the ICF [3,14,15,16,17]. Such an approach enables identification of disease-specific markers of problems with functioning, but also common cross-disease issues, i.e., the so called horizontal epidemiology approach.

The aim of the present study is to evaluate the coverage of disability measures used in recent research on primary headaches by mapping their content against the ICF classification, and to identify both issues that are specific to single primary headaches, namely, migraine, TTH, and CH, and issues that are common across the three disorders.

## 2. Materials and Methods

We searched SCOPUS for primary research papers, excluding literature reviews, letters, or commentaries, published from January 2015 to August 2020, in which any outcome measure that might reflect a disability or impact content was used in adult populations of patients with migraine, TTH, and CH. The search criteria were “headache OR migraine” combined with “disability OR impact” in records’ title and abstracts.

We limited our search to papers published in English and that were included in one of the following subject areas: Medicine, Multidisciplinary, Neuroscience, and Psychology. Specific filters were then applied to exclude publications that were clearly off topic (e.g., “Nuclear Magnetic Resonance Imaging”, “Physiology”, or “Animal Model”), or referred to non-adult populations (e.g., “Very Elderly” or “Preschool Child”), or that included conditions other than primary headaches (e.g., “Backache”, “Traumatic Brain Injury”, or “Cerebrovascular Accident”). For studies on medication overuse for headache, the primary headache was reported. Studies that did not make explicit the primary headache (for example studies on patients with “headache disorders” with no other specific reference), or those in which headache was referred as a symptom in the context of other conditions, were excluded.

The abstract and text of these records were then screened to identify the outcome measures that were employed which referred to disability or impact of primary headaches so that a comprehensive list could be compiled. Such a list was completed to show the use of different disability or impact outcome measures in the three main primary headache groups: migraine, TTH, or CH, including both episodic and chronic forms for each of them. We included questionnaires addressing disability and impact in terms of disability, work-related difficulties, and quality of life (QoL), excluding specific health problems which might be connected to primary headaches; examples of this included sleep problems, symptoms of depression, and anxiety.

Once the list of outcome measures used in these studies had been filled in, the content of these measures was mapped against ICF categories using established linking rules [18], with a minor variation. We decided to report the linkage not to the most precise ICF category, but to second-level ones. Such a choice was made with the intent to rely on clearer and more exploitable ICF categories. For example, presence of head pain was reported with the ICF category b280-Pain, and not with b28010-Pain in head and neck.

Results are reported at item, questionnaire, and overarching questionnaire level, showing the distribution of ICF categories also by ICF chapters (e.g., mental functions which is a higher level category for b134-Sleep problems and b152-Emotional problems) and domains, namely BF, BS, A&P, and EF.

Second, an overview of the amount of linked categories by ICF domain and by primary headache was carried out. As the amount of linked ICF categories is dependent on the number of assessment instruments used in each condition, and considering the possible bias due to the fact that a single ICF category can be linked to multiple concepts, to represent the amount of categories in each condition and domain the single categories were counted only once. For example, the ICF category b130-Energy and drive can be represented with multiple concepts; in the SF-36, it was linked to three different items (“Did you have a lot of energy?”, “Did you feel worn out?”, and “Did you feel tired?”), that refer to two different concepts, i.e., being full of energy vs. being tired or worn-out. To come back to the aforementioned example, the ICF category b130 was linked on 17 occasions to 14 different assessments, including the SF-36, which was used in all of the three headache types. In order to represent the span of information for each domain, the amount of single categories was reported as a percentage of the total second-level ICF categories.

Finally, a description of the most commonly used assessment instruments and their coverage is made for each primary headache, highlighting both disease-specific and common elements addressed by the assessment instruments. An element of functioning was defined as specific to the assessment instruments of one primary headache if the corresponding ICF category was covered in one-third of the assessment instruments used in that condition. Those elements that resulted as functioning elements specific to all the three primary headache forms included in the study were defined as common elements.

## 3. Results

### 3.1. Overview of Literature Search

**Figure 1:**
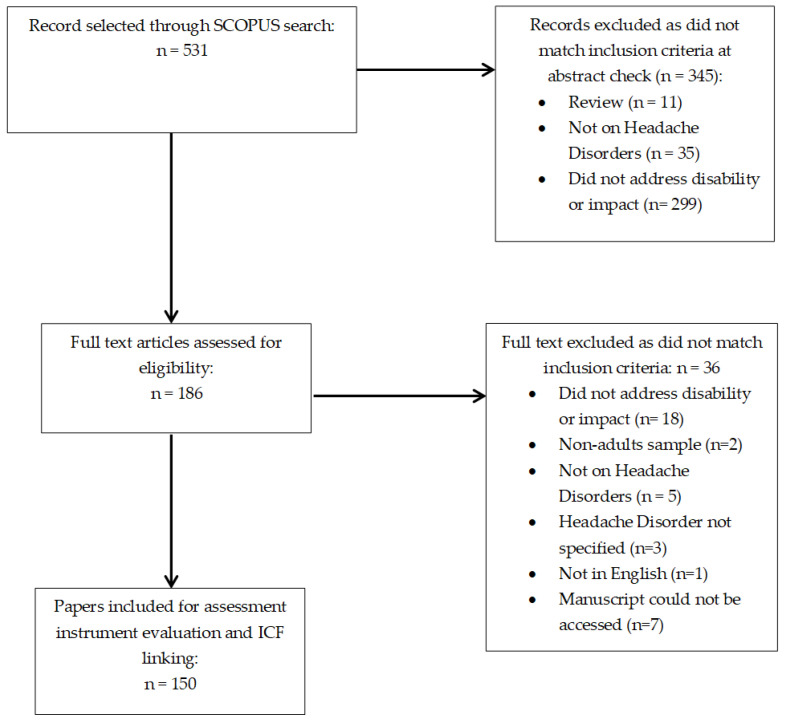
Flow-chart of selected studies.

A total of 531 records were screened for the presence of disability or impact outcome measures in the adult population of patients with primary headaches. Of them, 345 were excluded at abstract check, whereas 186 contained information on the presence of disability or impact measures in patients with primary headache, and their full-texts were therefore analyzed. A further 36 papers were excluded at full-text analysis: 18 because no information on disability or impact measure was included; two because on non-adult populations; five because the main condition was not migraine, TTH, or CH; three because the primary headache was not specified; one because it was not in English; seven because the manuscript could not be accessed, despite the corresponding authors were sent e-mail messages asking for a copy of their paper. Figure 1 shows the flow-chart of papers’ selection.

In total, 150 papers were included [19,20,21,22,23,24,25,26,27,28,29,30,31,32,33,34,35,36,37,38,39,40,41,42,43,44,45,46,47,48,49,50,51,52,53,54,55,56,57,58,59,60,61,62,63,64,65,66,67,68,69,70,71,72,73,74,75,76,77,78,79,80,81,82,83,84,85,86,87,88,89,90,91,92,93,94,95,96,97,98,99,100,101,102,103,104,105,106,107,108,109,110,111,112,113,114,115,116,117,118,119,120,121,122,123,124,125,126,127,128,129,130,131,132,133,134,135,136,137,138,139,140,141,142,143,144,145,146,147,148,149,150,151,152,153,154,155,156,157,158,159,160,161,162,163,164,165,166,167,168], in which 26 assessment instruments were used: 15 addressed disability or impact, defined in terms of interference of headache with daily functioning, two addressed work-related difficulties, and nine addressed QoL. Table 1 shows the use of the different assessment instruments across the three main primary headaches. The most commonly used assessments were MIDAS [6], used in 86 studies, HIT-6 [7], used in 71 studies, and the MSQ [8], used in 28 studies. Taken together, 338 single items were included in the 26 assessments, and a total of 432 linkages to 69 ICF categories were made. In addition to this, 69 items could not be linked because not covered or because the content was not sufficiently defined.

**Figure 2:**
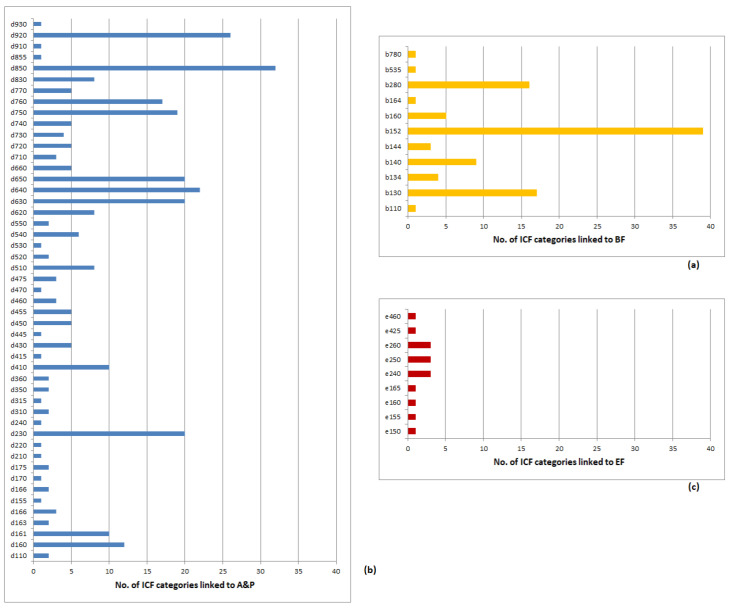
Overview of linked ICF categories for the three primary headaches. (**a**) Body Functions, (**b**) Activities and Participation, (**c**) Environmental Factors.

Table 2 reports the distribution of ICF-linked items for each assessment instrument and for each ICF domain, and shows that disability and impact assessment instruments were linked to BF, A&P, and EF domains, but not to BS. Please see Supplementary Materials Table S1 for item by item-level ICF-linked categories by domain for each assessment instrument). Figure 2 provides an overview of linked ICF categories for the three primary headaches together.

The most represented domain was A&P, with 49 different categories (corresponding to 50.5% of the 97 specific second-level A&P categories) and 320 single links of which the most represented were d850-Remunerative employment (32 single links), d920-Leisure activities (26 single links), d640-Doing housework (22 single links), d-230-Carrying out daily routing, d630-Preparing meals, and d650-Caring for household objects, each with 20 single links.

The second most represented domain was BF, with 11 different categories (corresponding to 12.5% of the 88 specific second-level BF categories) and 97 single links, of which the most represented being b152-Emotional functions (39 single links), b130-Energy level (17 single links), and b280-Pain (16 single links).

The third domain was that of EF, with 9 categories linked (corresponding to 14.1% of the 64 specific second-level EF categories) and 15 single links, of which the most represented were e240-Light, e250-Sound, and e260-Air quality with two single links each.

**Figure 3:**
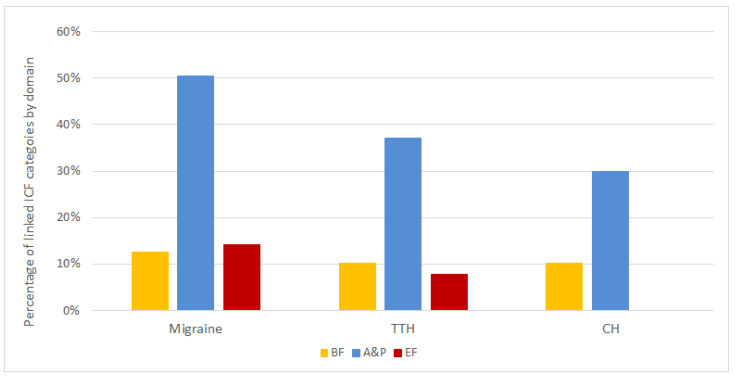
Overview of the percentage of linked categories by ICF domain and by each primary headache.

Figure 3 provides an overview of the amount of linked categories by ICF domain and by each primary headache. The coverage was similar with regard to BF, and comprised between 10% and 13%, whereas differences can be appreciated for A&P, which were much more described by the assessments used for migraine (51% of ICF categories were linked to assessment instruments) than by the assessments used for TTH and CH (37% and 30% respectively). Differences also were found for EF, with a percentage of linked ICF categories of 14% for migraine, 8% for TTH, and zero for CH.

### 3.2. Assessment Instruments Used in Migraine, TTH, and CH Research

Almost all assessment instruments retrieved in this review have been used in migraine research, with the exclusion of two CH-specific assessments.

The most used tools were MIDAS [6], HIT-6 [7], and the MSQ [8], used in 80, 56, and 27 studies, respectively. In addition to this, 11 studies employed an instrument from the WHO Disability Assessment Schedule (WHODAS) family [169,170] (either the 36 or 12 item version), and 10 studies employed an instrument from the Short-Form (SF) Health Survey family, i.e., the SF-36, SF-12, or SF-8 [171]. The ICF-linked items by domain for each of the assessment tools used in the research papers included in our analysis are reported in Table 2.

In terms of content coverage, MIDAS and HIT-6 have similar content, which covers difficulties with education or employment, difficulties with home-based activities, and difficulties with leisure time, with the HIT-6 also addressing mental functions connected to energy level and emotional functions. The same content is covered by the MSQ, with the addition of difficulties with informal and family relations.

Instruments derived from the WHODAS and from the SF Health Survey families were used in fewer studies. The results of our linking exercise analyzed the 36 items version of these questionnaires. These two instruments were linked to a higher amount of ICF categories. In addition to the aforementioned issues, i.e., difficulties with education or employment, with home-based activities, with leisure time, and difficulties with informal and family relations, the SF-36 also covers issues connected to mobility and self-care. The WHODAS 2.0 content further covers issues connected to communication activities, expands the scope of relations, and also included some EFs that may hinder patients’ ability to perform activities, or worsen headache. The main reason for such a wider variety of content lies in the fact that these two measures are not specific for headache disorders and cover also how much the environment could be a barrier for headache patients.

Two instruments were used to specifically address work-related difficulties in patients with migraine, the WPAI [172] in three studies and the HEADWORK questionnaire [110] in two studies, which were specifically developed on samples of patients with episodic and chronic migraine. The first addressed difficulties in work-related activities in general, whereas in the second instrument work-related problems were broken down in a variety of single tasks, and also included few EF that may hinder work-related tasks or worsen headache.

Other less used tools were the Migraine Functional Impact Questionnaire (MFIQ) [68], the Migraine Physical Function Impact Diary (MFIPD) [124] addressing impact on daily functioning, and the 24-h Migraine Quality of Life Questionnaire (24-h MqoLQ), addressing various dimensions of QoL [173,174]. Finally, the HEADWORK questionnaire [110] was validated on migraine patients, although deemed to be feasible to address work-related problems of other headache patients.

In TTH research, the most used tools were the HIT-6, the Headache Disability Inventory (HDI) [175] and the SF-36, used in eight, six, and four studies, respectively.

The HIT-6 content covers difficulties with education or employment, difficulties with home-based activities, difficulties with leisure time, and mental health issues connected to energy levels and emotional functions. The HDI includes HIT-6 content, but also issues connected to cognitive functions, muscle tenderness, transportation, and difficulties with relations. Finally, the SF-36 content covers difficulties with education or employment, with home-based activities, mobility, self-care, leisure time, and difficulties with informal and family relations.

It has to be noted that no TTH-specific measure has been developed, and that research on TTH is based on measures developed for migraine (i.e., MIDAS [6] and the MSQ [8]) or designed for headache disorders as a whole group (i.e., HIT-6 [7] or the HDI [175]).

The most used assessment instruments in CH were the HIT-6 and the SF-36, used in seven and four studies respectively. Two CH-specific assessment instruments have been developed, the Cluster Headache Quality of life scale (CHQ) [135], and the Cluster Headache Scales (CHS) [56]. The CHQ content covers mental functions connected to impulse control, sleep, cognitive, and emotional functions, as well as pain, and a set of activities connected to watching, reading, self-care, relations, general daily life, work, and leisure time. The CHQ also covers some EF, specifically crowded and noisy places. The CHS is intended to address CH impact, and it covers mental health issues connected to energy level and mood, as well as impact on daily live and leisure time.

### 3.3. Common and Disease-Specific Elements of Functioning Covered by the Assessment Instruments

Table 3 shows common and disease specific elements of functioning covered by the assessment instruments used in the three primary headaches. The one-third threshold corresponded to six assessment instruments for migraine and three for both TTH and CH.

There were 15 ICF categories whose content was covered in at least one-third of the assessments used in each of the three primary headaches. Half of them corresponded to the core issues addressed by the most commonly used assessment, i.e. impact on daily life activities (d230-Carrying out daily routine), on home work (d620-Acquisition of goods and services; d630-Preparing meals; d640-Doing housework; d650-Caring for household objects), on school and work-related tasks (d830-Higher education; d850-Remunerative employment), and on leisure time (d920-Recreation and leisure). The remaining A&P categories refer to personal relations (d750-Informal social relationships; d760-Family relationships) and to attention-related tasks (d160-Focusing attention; d161-Directing attention). In addition to this, three BF were included: pain (b280), emotional functions (b152), which covered items connected to the expression of depression, anxiety, and anger, and the ICF category b130-Energy and drive, whose content includes both energy level (i.e. being full of energy and being tired) and impulse control. Minor differences existed, considering the fact that the instruments used in migraine research did not commonly address self-care issues such as washing and dressing.

## 4. Discussion

The results of this analysis show that the assessment instruments commonly used in recent research on disability and impact of migraine cover a wide variety of body function impairments, simple and complex activities, as well as a set of few environmental factors. In total, 26 different instruments were found, mostly addressing disability and impact. However, despite such variability, the vast majority of published research was carried out with a few assessment instruments, which cover only few domains of functioning, specifically the impact on daily life activities, homework, school and work-related tasks, leisure time, informal and family relations, as well as on symptoms such as pain, emotional difficulties, energy level, and impulse control. Conversely, the role of environmental factors, including the most known triggers or exacerbating factors, is poorly considered.

There are some critical aspects that pertain to the assessment instruments that were found in our review. First, there is scarce consideration of elements connected to the physical and social environment, which is critical in consideration of the environment-related hindrances that people with headache disorders, and migraine in particular, experience. Migraine is in fact associated to symptoms like photophobia, phonophobia, osmophobia, and nausea [176,177]: therefore, light, noise and smell, as well as being in a crowded place are commonly avoided by people with migraine, and constitute core elements of most of daily life, at work as well as at home. Despite this, such elements are not accounted for in most assessment instruments, and therefore in most of the research on headache disorders. Second, much of the assessment instruments that are used in TTH and CH research have been developed for migraine. This hinders research on CH, as the outcome measures used to address impact of migraine focus on the "frequency of occasions" in which a patient was unable to do something, which is the focus of MIDAS, or the "degree" to which a patient was unable to do something which is the focus of HIT-6 and HDI. Such a perspective on impact assessment is inappropriate for evaluation of CH, as the outcomes of interest for CH include frequency, intensity, duration of attacks, and time to relief [10], as well as the whole set of behavioral issues associated with CH, including sleep disturbance, depression, anxiety, suicidal ideation and behavior, aggressiveness, and cognitive deficits [178,179]. These behavioral aspects are better accounted for by two recently developed CH-specific assessment instruments, namely, the CHS and the CHQ [56,135]. Should these two PROS have the same impact for CH research that MIDAS, HIT-6, and the MSQ have had on migraine research, it can be expected that future research addressing CH impact will be improved. Third, several instruments merge a variety of concepts into a single question which can be confusing for patients and weakens the scope of an assessment instrument. Item development should on the contrary include a simple and direct question addressing one concept only [180].

Other problems in the analysis are related to issues that we could not retrieve in our work. First, there is a paucity of studies addressing the impact of headache disorders on work-related activities. Some literature on this topic, and specifically on migraine and chronic migraine, exists and it shows that headache disorder impact is a function of headache frequency, treatment response and features of work itself [27,30,43,44,110,181,182]. However, such a kind of research t is completely lacking for TTH and CH. However, as also shown in another review [5], this kind of research is mostly focused on the amount of lost workdays, which follows a MIDAS-like approach. This information can be useful in estimating the economic burden of migraine, but becomes less useful if a closer description of the set of difficulties with specific work-related tasks is desired. Again, this information is of clear utility if tailored solutions are to be planned for single patients, but also in order to assess the impact of different treatments on work-related activities outcomes. A specific assessment instrument, the HEADWORK questionnaire was developed on a large set of patients with episodic and chronic migraine [43,110], and it also worked for patients with TTH. HEADWORK can be fruitfully employed to address the impact of triggers and of treatments on work-related difficulties, thus contributing to evaluate the cost-effectiveness of different treatments.

Second, no TTH-specific assessment instrument was retrieved, and all research on TTH is based on instruments developed for migraine. The problem with this research approach is that the features of migraine include clinical aspects that are not present in TTH, with the risk of getting information about symptoms and disease impact that may not match the experience of patients with TTH (e.g. impact on concentration, need of lying down, problems with light, noise, or nausea). Whether the opposite situation, i.e., that items covering disability or QoL issues that are specific to TTH only are missing, cannot be ascertained. To get to this point TTH-specific assessment instruments should therefore be developed moving from the ground of patients’ experience with TTH, with specific item-generation approaches.

Finally, the scope of information obtained through single assessment instruments is limited to few domains. Our analysis showed which domains of functioning are mostly covered by the assessment instruments used in headache research, demonstrating that 15 domains are common to the three primary headache disorders, but only eight out of the 26 assessment instruments covered 15 or more domains of functioning. Instruments are needed that enable a full appraisal of the impact of headache disorders on patients’ daily lives and on EFs on their headaches and lives, and not only on a restricted set of areas. This would not be of secondary relevance, as headache disorders are considered as severely disabling disorders, specifically due to their high prevalence. The burden of migraine has been recognized as impact on several domains of life, including inter-ictal phases, whereas the burden of TTH is probably underrated, as large studies addressing the impact of TTH are lacking. This is also the conclusion of a study based on GBD 2016 estimates for migraine and headache [183]. The short supply of data is a problem for the production of estimates, as large-scale epidemiological studies on headache disorders are not performed in many parts of the world, and data on headache disorders are poorly captured in national health surveys and in administrative datasets. Improving the quality of data collection and the scope of reported information, and planning regular large-scale surveys could help produce stronger evidence of the need for action on headaches. This is of relevance as headache disorders are important causes of disability worldwide, and therefore deserve greater attention and research resource allocation [183]. In our opinion, the first step that needs to be made is to increase the knowledge on the impact of TTH and of CH, using both disease-specific and generic instruments (e.g., the WHODAS 2.0), as this is a viable way to inform the estimates on disability associated with TTH and CH.

Some limitations have to be acknowledged. First, we relied on SCOPUS only, rather than on a wider set of search engines, which might have caused a loss in studies’ identification. However, it has to be remembered that our focus was not on research results, but on the assessment instruments that have been used. In this sense, the number of studies that we identified has likely been sufficient to provide an overview of instruments’ use. Second, we were unable to locate some studies, despite requests that were sent to the corresponding authors. Third, our decision to link assessments’ items to second-level ICF categories necessarily resulted in a partial loss of information. For example, the ICF category b130-Energy and Drive was linked to two different concepts, vitality/energy and lack of energy/tiredness, which should have been linked to the third-level category b1300-Energy level, and loss of control/impulsivity, which should be linked to the to the category b1304-Impulse control. However, this choice had the advantage of relying on ICF categories that are much clearer for the purpose of defining the content of the assessment instruments. Finally, is has to be considered that this review took into account adult populations and, therefore, adult assessment instruments, but headache disorders are prevalent among pediatric populations as well, with significant impact on QoL and the ability to perform school and non-school activities [184,185].

## 5. Conclusions

In conclusion, we performed a literature search and analysis aimed at addressing the most commonly used assessment instruments for impact in headache disorders and mapped their content against the ICF classification. A total of 26 different assessment instruments were retrieved, the most commonly used being MIDAS, HIT-6, and the MSQ, and the whole set of instruments was linked to 69 ICF categories. The most commonly linked ICF categories from A&P domain were d850-Remunerative employment, d920-Leisure activities, d640-Doing housework, d230-Carrying out daily routing, d630-Preparing meals, and d650-Caring for household objects. The most commonly linked ICF categories from BF domain were b152-Emotional functions, b130-Energy level, and b280-Pain.

This review shows that most of the information that is available to describe headaches’ impact is in few domains of functioning, and that EF are generally not addressed. Most of the research is based on instruments that were developed for migraine, which is critical in particular for CH, and the impact of headache disorders on work-related activities has been poorly evaluated.

Further research is needed to expand the scope of headaches impact on daily life activities, and to raise knowledge for the less represented areas of headache disorders, including large-scale studies on TTH impact.

## Figures and Tables

**Table 1:** Use of different assessment instruments across the three main primary headache disorders.

Assessment Instruments	Primary Headache		
	Migraine	TTH	CH
**Disability Assessments**			
MIDAS	N = 80 [19,21,25,26,28,29,30,31,32,36,37,38,39,40,41,42,44,46,47,48,50,52,54,55,57,63,64,65,66,67,69,70,71,73], [75,76,77,80,81,82,84,85,88,89,90,91,92,94,98,99,102,103,105,106,108,109,110,116,118,119,125], [130,132,133,136,141,142,144], [147,148,149,154,155,156,157,159,162,164,165,167]	N = 4 [94,105,112,133]	N = 2 [126,128]
HIT-6	N = 56 [22,23,29,32,33,34,35,39,43,46,50,51,52,58,62,68,74,75,83,89,92,93,96,97,100,101,103,104,106,107,111,114,115,116,117,118], [121,124,131,132,137,139,140,142,143], [146,147,148,151,152,153,157,160,161,166,168]	N = 8 [35,45,93,97,111], [115,161,166]	N = 7 [20,59,61,86,87], [126,128]
HDI	N = 6 [63,93,123,137,138,158]	N = 6 [24,78,79,93,120,129]	N = 1 [56]
WHODAS 2.0; WHODAS-12	N = 11 [36,93,95,110,127,130,155], [156,158,164,165]	N = 1 [93]	
HURT	N = 3 [72,82,133]	N = 2 [72,133]	
PDI	N = 2 [81,168]		
PROMIS-PI 6a; PROMIS-PI 6b	N = 2 [44,75]		
PROMIS-PF 10a	N = 2 [68,124]		
NDI		N = 1 [45]	
HDQ	N = 1 [93]	N = 1 [93]	
CHS			N = 1 [56]
MFIQ	N = 1 [68]		
MPFID	N = 1 [124]		
**Work-related difficulties assessments**			
WPAI	N = 3 [27,30,44]		
HEADWORK	N = 2 [43,110]		
**Quality of life assessments**			
MSQ	N = 27 [34,36,38,53,68,69,75,92,96,100,103], [110,116,119,124,125,127,130,139,147], [148,154,155,156,158,164,165]		N = 1 [135]
SF-36; SF-12; SF-8	N = 10 [34,42,60,97,121,122], [134,145,154,158]	N = 4 [97,112,120,122]	N = 4 [61,87,128,135]
EQ-5D-5L	N = 4 [34,74,113,168]		N = 2 [126,135]
CHQQ	N = 1 [133]	N = 1 [133]	
EUROHIS-QOL 8-item	N = 2 [72,150]	N = 2 [72,150]	
24-h MQoLQ	N = 1 [49]		
CHQ			N = 1 [135]

Note. TTH, Tension-Type Headache; CH, Cluster Headache; MIDAS, Migraine Disability Assessment; HIT-6, 6-Item Headache Impact Test; HDI, HDI, Headache Disability Inventory; WHODAS, WHO Disability Assessment Schedule; HURT, Headache Under-Response to Treatment; PDI, Pain Disability Inventory; PROMIS-PI, Patient-Reported Outcomes Measurement Information System—Pain Interference; PROMIS-PF PROMIS—Physical Function; NDI, Neck Disability Index; HDQ, Headache Disability Questionnaire; CHS, Cluster Headache Scale; MFIQ, Migraine Functional Impact Questionnaire; MPFID, Migraine Physical Function Impact Diary; WPAI, Work Productivity and Activity Impairment Questionnaire; MSQ, Migraine-Specific Quality of Life Questionnaire; SF-36 (SF-12; SF-), 36-Items (12-Items; 8-Items) Short-Form Health Survey; CHQQ, Comprehensive Headache-related Quality of life Questionnaire; EUROHIS-QOL 8-item, European Health Interview Survey-Quality of Life 8-item index; 24-h MQoLQ, 24-Hour Migraine Quality of Life Questionnaire; CHQ, Cluster Headache Quality of life scale.

**Table 2:** The International Classification of Functioning, Disability, and Health (ICF)-linked categories by domain for each assessment instrument.

Assessment Instruments	ICF Domain			No. of Linked ICF Categories
	BF	A&P	EF	
**Disability assessments**				
MIDAS	b280	d620,d640,d650,d660,d750,d760,d830,d850,d920		10
HIT-6	b130,b152,b280,	d160,d230,d640,d650,d620,d830,d850,d920		11
HDI	b130,b152,b140,b160,b164,b780	d160,d161,d163,d166,d230,d470,d475,d730,d740,d750,d760,d920		18
WHODAS 2.0	b140,b144,b152	d160,d161,d155,d175,d315,d350,d410,d415,d450,d460,d510,d540,d550,d630,d640,d650,d720,d730,d750,d760,d770,d850,d910,d920,d930	e150,e155,e160,e165,e460	33
HURT		d630,d640,d650,d750,d760,d850,d830,d920		8
PDI		d550,d630,d640,d650,d660,d770,d850,d855,d920		9
PROMIS-PI 6a		d230,d630,d640,d650,d920		5
PROMIS-PI 6b	b140	d160,d161,d230,d430,d620,d630,d640,d650,d720,d730,d740,d750,d920		14
PROMIS-PF 10a;		d410,d430,d445,d450,d455,d510,d530,d540,d620,d640,d920		11
NDI	b134,b140,b280	d160,d161,d166,d430,d475,d510,d540,d850,d920		12
HDQ	b280	d630,d640,d650,d750,d760,d830,d850,d920		9
CHS	b130,b152	d230,d920		4
MFIQ	b130,b152	d160,d161,d230,d410,d510,d520,d620,d630,d640,d650,d660,d740,d750,d760,d770, d830,d850,d920	e240,e250,e260	23
MPFID	b140	d160,d161,d230,d410,d630,d640,d650,d710,d720		10
**Work-related difficulties assessments**				
WPAI		d230,d850		2
HEADWORK	b140	d160,d161,d166,d170,d175,d210,d220,d240,d310,d350,d360,d460,d475,d710,d720,d740,d750	e240,e250,e260,e425	22
**Quality of life assessments**				
MSQ	b130,b152	d160,d166,d230,d630,d640,d650,d750,d760,d850,d920		12
SF-36	b130,b152,b280	d410,d430,d445,d450,d455,d510,d540,d620,d630,d640,d650,d750,d760,d850,d920		18
EQ-5D-5L	b152,b280	d450,d510,d540,d630,d640,d650,d660,d830,d850,d920		12
CHQQ	b130,b140,b152,b160	d160,d161,d163,d630,d640,d650,d750,d760,d770,d850,d920		15
EUROHIS-QOL 8-item	b130	d230,d750,d760		4
24-h MQoLQ	b110,b130,b134,b152,b280,b535	d620,d640,d650,d710,d720,d730,d740,d750,d760,d830,d850	e240,e250	19
CHQ	b130,b134,b140,b144,b152,b160,b280	d110,d160,d161,d166,d230,d510,d520,d540,d750,d760,d770,d850,d920	e215,e250	22

Note. ICF, International Classification of Functioning, Disability and Health; BF, Body Functions; A&P, Activity and Participation; EF, Environmental Factors; MIDAS, Migraine Disability Assessment; HIT-6, 6-Item Headache Impact Test; HDI, HDI, Headache Disability Inventory; WHODAS, WHO Disability Assessment Schedule; HURT, Headache Under-Response to Treatment; PDI, Pain Disability Inventory; PROMIS-PI, Patient-Reported Outcomes Measurement Information System—Pain Interference; PROMIS-PF PROMIS—Physical Function; NDI, Neck Disability Index; HDQ, Headache Disability Questionnaire; CHS, Cluster Headache Scale; MFIQ, Migraine Functional Impact Questionnaire; MPFID, Migraine Physical Function Impact Diary; WPAI, Work Productivity and Activity Impairment Questionnaire; MSQ, Migraine-Specific Quality of Life Questionnaire; SF-36 (SF-12; SF-), 36-Items (12-Items; 8-Items) Short-Form Health Survey; CHQQ, Comprehensive Headache-related Quality of life Questionnaire; EUROHIS-QOL 8-item, European Health Interview Survey-Quality of Life 8-item index; 24-h MQoLQ, 24-h Migraine Quality of Life Questionnaire; CHQ, Cluster Headache Quality of life scale.

**Table 3:** Common and disease-specific elements of functioning covered by the assessment instruments.

ICF Domains and Categories	Primary Headache			Common to the Three Disorders
	Migraine	TTH	CH	
Body Functions				
b130-Energy and drive	√	√	√	√
b140-Attention	√	√		
b152-Emotional functions	√	√	√	√
b280-Pain	√	√	√	√
Activities and Participation				
d160-Focusing attention	√	√	√	√
d161-Directing attention	√	√	√	√
d166-Reading		√	√	
d230-Carrying out daily routine	√	√	√	√
d510-Washing oneself		√	√	
d540-Dressing			√	
d620-Acquisition of goods and services	√	√	√	√
d630-Preparing meals	√	√	√	√
d640-Doing housework	√	√	√	√
d650-Caring for household objects	√	√	√	√
d750-Informal social relationships	√	√	√	√
d760-Family relationships	√	√	√	√
d830-Higher education	√	√	√	√
d850-Remunerative employment	√	√	√	√
d920-Recreation and leisure	√	√	√	√
Total no. of ICF categories	16	18	18	15

Notes. √—ICF category covered by one-third of the instruments. TTH, Tension-Type Headache; CH, Cluster Headache.

## Data Availability

The data presented in this study are available in Supplementary materials (Table S1).

## Supplementary Materials

The following are available online at https://www.mdpi.com/1660-4601/18/1/246/s1, Table S1: Item by item-level ICF-linked categories by domain for each assessment instrument.
