# Supplementary material for: Mapping Assessments Instruments for Headache Disorders against the ICF Biopsychosocial Model of Health and Disability
Source: Int J Environ Res Public Health. 2020 Dec 31;18(1):246. doi: 10.3390/ijerph18010246 (PMC7795912; doi:10.3390/ijerph18010246)
Supplement: Supplementary file 1 [file ijerph-18-00246-s001.pdf]

Table S1. Item by item-level ICF-linked categories by domain for each assessment instrument

Assessment Instruments	BF	A&P	EF	Nd/NC
**DISABILITY ASSESSMENTS**				
MIDAS				
On how many days in the last 3 months did you miss work or school because of your headaches? / How many days in the last 3 months was your productivity at work or school reduced by half or more because of your headaches?		d830,d850		
On how many days in the last 3 months did you not do household work (such as housework, home repairs and maintenance, shopping, caring for children and relatives) because of your headaches? / How many days in the last 3 months was your productivity in household work reduced by half of more because of your headaches?		d640,d650,d620, d660		
On how many days in the last 3 months did you miss family, social or leisure activities because of your headaches?		d750,d760,d920		
On how many days in the last 3 months did you have a headache?	b280			
On average how painful were these headaches?	b280			
HIT-6				
When you have headaches, how often is the pain severe?	b280			
How often do headaches limit your ability to do usual daily activities including household work, work, school, or social activities?		d230,d640,d650, d620,d830,d850, d920		
When you have a headache, how often do you wish you could lie down?	b280,b130			
In the past 4 weeks, how often have you felt too tired to do work or daily activities because of your headaches?	b130	d850,d230		
In the past 4 weeks, how often have you felt fed up or irritated because of your headaches?	b152			
In the past 4 weeks, how often did headaches limit your ability to concentrate on work or daily activities?	b280	d160,d230		
HDI				
Do you feel disabled because of your headache?				nd
Do you feel restricted in performing your routine daily activities?		d230		
Do you feel no one understands the effect your headaches have on your life?				nc
Do you restrict your recreational activities (for example, sports, hobbies) because of		d920		

your headaches?				
Do your headaches make you angry?	b152			
Do you feel that you are going to lose control because of your headaches?	b130			
Are you less likely to socialize because of your headaches?		d730,d740,d750		
Do you feel like your spouse (or significant other), family and friends have no idea what you are going through because of your headaches?				nc
Do you feel your headaches are so bad that you will go insane?				nc
Is your outlook on the world affected by your headaches?				nc
Are you afraid to go outside when you feel a headache is starting?				nd
Do you feel desperate because of your headaches?	b152			
Are you concerned that you are paying penalties at work or at home because of headaches?				nd
Do your headaches place stress on your relationships with family or friends?		d750,d760		
Do you avoid being around people when you have a headache?		d750		
Do you believe your headaches are making it difficult for you to achieve your goals in life?				nc
Are you unable to think clearly because of your headaches?	b160			
Do you get tense (for example, muscle tension) because of your headaches?	b780			
Do you not enjoy social gatherings because of your headaches?		d750		
Do you feel irritable because of your headaches?	b152			
Do you avoid traveling because of your headaches?		d470/d475		
Do your headaches make you feel confused?	b164	d163		
Do your headaches make you feel frustrated?	b152			
Do you find it difficult to read because of your headaches?		d166		
Do you find it difficult to focus your attention away from your headaches and on other things?	b140	d160/d161		
WHODAS 2.0; WHODAS-12				
Concentrating on doing something for ten minutes?	b140	d160/d161		
Remembering to do important things?	b144			
Analysing and finding solutions to problems in day to day life?		d175		
Learning a new task, for example, learning how to get to a new place?		d155		
Generally understanding what people say?		d315		
Starting and maintaining a conversation?		d350		
Standing for long periods such as 30 minutes?		d415		

Standing up from sitting down?		d410		
Moving around inside your home?		d460		
Getting out of your home?		d460		
Walking a long distance such as a kilometre [or equivalent]?		d450		
Washing your whole body?		d510		
Getting dressed?		d540		
Eating?		d550		
Staying by yourself for a few days?				nd
Dealing with people you do not know?		d730		
Maintaining a friendship?		d750		
Getting along with people who are close to you?		d760		
Making new friends?		d720		
Sexual activities?		d770		
Taking care of your household responsibilities?		d630/d640/d650		
Doing your most important household tasks well?		d630/d640/d650		
Getting all the household work done that you needed to do?		d630/d640/d650		
Getting your household work done as quickly as needed?		d630/d640/d650		
Your day to day work?		d850		
Doing your most important work tasks well?		d850		
Getting all the work done that you need to do?		d850		
Getting your work done as quickly as needed?		d850		
How much of a problem did you have joining in community activities (for example, festivities, religious or other activities) in the same way as anyone else can?		d910,d930		
How much of a problem did you have because of barriers or hindrances in the world around you?			e150,e155,e160	
How much of a problem did you have living with dignity because of the attitudes and actions of others?			e460	
How much time did you spend on your health condition, or its consequences?				nc
How much have you been emotionally affected by your health condition?	b152			
How much has your health been a drain on the financial resources of you or your family?			e165	
How much of a problem did your family have because of your health problems?				nc
How much of a problem did you have in doing things by yourself for relaxation or pleasure?		d920		

HURT				
On how many days in the last month did you have a headache?				nc
On how many days in the last three months did your headaches make it hard to work, study or carry out household work?		d850,d830, d630/d640/d650		
On how many days in the last three months did your headaches spoil or prevent your family, social or leisure activities?		d760,d750,d920		
On how many days in the last month did you take medication to relieve a headache? (Do not count preventative medication.)				nc
When you take your headache medication, does one dose get rid of your headache and keep it away?				nc
Do you feel in control of your headaches?				nc
Do you avoid or delay taking your headache medication because you do not like its side-effects?				nc
What have you been told is your headache diagnosis?				nc
Do you feel you understand this diagnosis?				nc
PDI				
Family/home responsibilities: This category refers to activities of the home or family. It includes chores or duties performed around the house (eg, yard work) and errands or favors for other family members (eg, driving the children to school).		d630,d640,d650, d660		
Recreation: This category includes hobbies, sports, and other similar leisure time activities.		d920		
Social activity: This category refers to activities that involve participation with friends and acquaintances other than family members. It includes parties, theater, concerts, dining out, and other social functions.		d920		
Occupation: This category refers to activities that are a part of or directly related to one's job. This includes nonpaying jobs as well, such as that of a housewife or volunteer worker.		d850,d855		
Sexual behavior: This category refers to the frequency and quality of one's sex life.		d770		
Life-support activity: This category refers to basic life-supporting behaviors such as eating, sleeping, and breathing.		d550		
PROMIS-PI 6a				
How much did pain interfere with your day to day activities?		d230		
How much did pain interfere with work around the home?		d630		
How much did pain interfere with your ability to participate in social activities?		d920		

How much did pain interfere with your household chores?		d630,d640,d650		
How much did pain interfere with the things you usually do for fun?		d920		
How much did pain interfere with your enjoyment of social activities?		d920		
PROMIS-PI 6b				
How much did pain interfere with your enjoyment of life?				nd
How much did pain interfere with your ability to concentrate?	b140	d160,d161		
How much did pain interfere with your day to day activities?		d230		
How much did pain interfere with your enjoyment of recreational activities?		d920		
How much did pain interfere with doing your tasks away from home (e.g., getting groceries, running errands)?		d620, d630,d640,d650, d430		
How often did pain keep you from socializing with others?		d720/d730/d740/ d750		
PROMIS-PF 10a;				
Does your health now limit you in doing vigorous activities, such as running, lifting heavy objects, participating in strenuous sports?		d455,d430,d920		
Does your health now limit you in walking more than a mile (1.6 km)?		d450		
Does your health now limit you in climbing one flight of stairs?		d455		
Does your health now limit you in lifting or carrying groceries?		d620,d430		
Does your health now limit you in bending, kneeling, or stooping?		d410		
Are you able to do chores such as vacuuming or yard work?		d445,d640		
Are you able to dress yourself, including tying shoelaces and buttoning your clothes?		d540		
Are you able to shampoo your hair?		d510		
Are you able to wash and dry your body?		d510		
Are you able to sit on and get up from the toilet?		d410,d530		
NDI				
Pain Intensity	b280			
Personal Care (Washing, Dressing, etc.)		d510,d540		
Lifting		d430		
Reading		d166		
Headaches	b280			
Concentration	b140	d160,d161		
Work		d850		

Driving		d475		
Sleeping	b134			
Recreation		d920		
HDQ				
How would you rate the usual pain of your headache on a scale from 0 to 10?	b280			
When you have headaches, how often is the pain severe?	b280			
On how many days in the last month did you actually lie down for an hour or more because of your headaches?	b280			
When you have a headache, how often do you miss work or school for all or part of the day?		d830,d850		
When you have a headache while you work (or school), how much is your ability to work reduced?		d830,d850		
How many days in the last month have you been kept from performing housework or chores for at least half of the day because of your headaches?		d630,d640,d650		
When you have a headache, how much is your ability to perform housework or chores reduced?		d630,d640,d650		
How many days in the last month have you been kept from non-work activities (family, social or recreational) because of your headaches?		d750,d760,d920		
When you have a headache, how much is your ability to engage in non-work activities (family, social or recreational) reduced?		d750,d760,d920		
CHS				
I am satisfied with my medical treatment.				nc
My medical treatment is effective.				nc
I tolerate my cluster headache medication well.				nc
My acute medication is effective.				nc
I suffer from the side effects of my medication.				nc
My acute medication for cluster headache causes side effects.				nc
My prophylaxis medication for cluster headache causes side effects.				nc
I am afraid that the disorder could get worse.				nc
I am afraid of a cluster attack.	b152			
I panic when I think about the next cluster attack.	b152			
I am worried about the next cluster attack.	b152			
The disease determines my life.				nc
I feel limited in everyday life.		d230		

The disease dominates my daily routine.		d230		
I withdraw socially due to my disease.		d920		
I am aggressive in a cluster attack.	b130			
In cluster attacks I hurt myself.				nc
I am impulsive in cluster attacks.	b130			
I attempted suicide.				nc
I have thoughts of suicide during a cluster attack.	b152			
MFIQ				
How often did migraine limit your ability to move your head		d410		
How often did migraine limit your ability to move your body		d410		
How often did migraine limit your usual activities that required physical effort				nd
How often did you feel that you needed to rest or lie down	b130			
How often did you feel too tired to do things?	b130			
How difficult was it to make yourself presentable (brushing hair, shaving, make-up)		d510,d520		
How often did migraine affect your daily routing or schedule		d230		
How often did you have to change your plans		d230		
How difficult was it to do your usual chores at home (tidying up, cleaning, preparing a meal, doing minor repairs)		d630,d640,d650		
How often did migraine limit your ability to do your usual chores outside home		d620		
How often did migraine affect your ability to do your usual work or school activities		d850,d830		
How often did migraine affect your ability to take care of family		d660		
How difficult was it for you to do activities that required you to concentrate		d160,d161		
How difficult was it to do activities in the presence of loud noise, strong smell or bright lights			e250,e260,e240	
How much did migraine affect your usual activities		d230		
How much did migraine affect your usual social interactions with family, friends or coworkers		d760,d750,d740		
How difficult was it for you to do activities with family or friends		d760,d750		
How much did you have to limit your social activities				nd
How often did you avoid being around other people				nd
How often did migraine prevent you from being intimate with your partner or spouse		d770		
How often did migraine limit your usual leisure activities		d920		

How frustrated did you feel about being unable to do what you needed to do	b152			
How often did you worry about your migraines	b152			
How often did you feel like a burden on others				nc
How often did you feel you lacked control of your life				nc
How disappointed did you feel about having migraine	b152			
MPFID				
Household chores		d630,d640,d650		
Activities outside the home				nd
Daily routine		d230		
Concentrate	b140	d160,d161		
Getting ready for day				nd
Avoid interaction		d710,d720		
Rest period				nd
Overall difficulty with usual activities				nd
Difficulty moving head		d410		
Difficulty moving body		d410		
Get out of bed		d410		
Bend over		d410		
Activities requiring physical effort				nd
**WORK-RELATED DIFFICULTIES ASSESSMENTS**				
WPAI				
Are you currently employed (working for pay)?		d850		
During the past seven days, how many hours did you miss from work because of problems associated with your MIGRAINE? Include hours you missed on sick days, times you went in late, left early, etc., because of your MIGRAINE.		d850		
During the past seven days, how many hours did you miss from work because of any other reason, such as vacation, holidays, time off to participate in this study?		d850		
During the past seven days, how many hours did you actually work?		d850		
During the past seven days, how much did your MIGRAINE affect your productivity while you were working?		d850		
During the past seven days, how much did your MIGRAINE affect your ability to do your regular daily activities, other than work at a job?		d230		
HEADWORK				
Paying attention to work tasks	b140	d160,d161		

Solving organizational problems at work		d175		
Starting a new work task		d210,d220		
Dealing with work problems		d240		
Reading and writing		d166,d170		
Using the PC		d360		
Answering the phone		d360,d310		
Driving a car		d475		
Moving from one place to another		d460		
Talking and interacting with other people		d350,d710,d720, d740,d750		
Understanding what is said		d310		
Noise in the workplace			e250	
Smell in the workplace			e260	
Brightness of workplace			e240	
Extended working hours				nd
Negative attitudes of colleagues			e425	
Air conditioning			e260	
**QUALITY OF LIFE ASSESSMENTS**				
MSQ				
have migraines interfered with how well you dealt with family, friends and others who are close to you?		d750,d760		
have migraines interfered with your leisure time activities, such as reading or exercising?		d920,d166		
have you had difficulty in performing work or daily activities because of migraine symptoms?		d850,d230 d630,d640,d650		
did migraines keep you from getting as much done at work or at home?		d850, d630,d640,d650		
did migraines limit your ability to concentrate on work or daily activities?		d160,d230,d850		
have migraines left you too tired to do work or daily activities?	b130	d230,d850		
have migraines limited the number of days you have felt energetic?	b130			
have you had to cancel work or daily activities because you had a migraine?		d850,d230		
did you need help in handling routine tasks such as every day household chores, doing necessary business, shopping, or caring for others, when you had a migraine?		d230,d630,d640, d650		
did you have to stop work or daily activities to deal with migraine symptoms?		d850,d230		

were you not able to go to social activities such as parties, dinner with friends, because you had a migraine?		d760,d920		
have you felt fed up or frustrated because of your migraines?	b152			
have you felt like you were a burden on others because of your migraines?				nc
have you been afraid of letting others down because of your migraines?				nc
SF-36;SF-12;SF-8				
In general, would you say your health is				nd
Compared to one year ago, how would you rate your health in general now?				nd
Vigorous activities, such as running, lifting heavy objects, participating in strenuous sports		d455,d430,d920		
Moderate activities, such as moving a table, pushing a vacuum cleaner, bowling or playing golf		d445,d920,		
Lifting or carrying groceries		d430		
Climbing several flights of stairs		d455		
Climbing one flight of stairs		d455		
Bending, kneeling or stooping		d410		
Walking more than a mile		d450		
Walking several blocks		d450		
Walking one block		d450		
Bathing or dressing yourself		d510,d540		
Cut down on the amount of time you spent on work or other activities (due to physical health)		d850		
Accomplished less than you would like (due to physical health)				nc
Were limited in the kind of work or other activities (due to physical health)		d850		
Had difficulty performing the work or other activities (for example, it took extra effort) (due to physical health)		d850		
Cut down the amount of time you spent on work or other activities (due to emotional problems)	b152	d850		
Accomplished less than you would like (due to emotional problems)				nc
Didn’t do work or other activities as carefully as usual (due to emotional problems)	b152	d850		
During the past 4 weeks, to what extent has your physical health or emotional problems interfered with your normal social activities with family, friends, neighbors or groups?	b152	d750,760,d920		
How much bodily pain have you had during the past 4 weeks?	b280			

During the past 4 weeks, how much did pain interfered with your normal work (including both work outside the home and housework)?	b280	d850, d620,d630,d640, d650		
Did you feel full of pep?	b152			
Have you been a very nervous person?	b152			
have you felt so down in the dumps that nothing could cheer you up?	b152			
have you felt calm and peaceful?	b152			
Did you have a lot of energy?	b130			
Have you felt downhearted and blue?	b152			
Did you feel worn out?	b130			
Have you been a happy person?	b152			
Did you feel tired?	b130			
During the past 4 weeks, how much of the time has your physical health or emotional problems interfered with your social activities (like visiting with friends, relatives etc)?		d920		
I seem to get sick a little easier than other people				nc
I am as healthy as anybody I know				nc
I expect my health to get worse				nc
My health is excellent				nc
EQ-5D-5L				
Mobility (i.e. problems in walking about)		d450		
Self-care (i.e. problems washing or dressing myself)		d510,d540		
Usual activities (e.g. work, study, housework, family or leisure activities)		d850,d830,d630, d640,d650,d660, d920		
Pain/Discomfort	b280			
Anxiety/Depression	b152			
CHQQ				
Work performance		d850		
Household chores		d630,d640,d650		
Social life		d750		
Leisure activities		d920		
Vacations/awaydays		d920		
Physical health				nc

Appearance				nc
Relationship with other family members		d760		
Sexual life		d770		
Sleep	b134			
Energy	b130			
Mood	b152			
Memory	b140			
Concentration	b140	d160,d161		
Thinking	b160	d163		
General health perceptions				nc
Irritability	b152			
Frustration	b152			
Abortive medication use				nc
Financial situation				nc
Embarrassment due to headaches				nc
Worries about headache	b152			
Life enjoyment				nc
EUROHIS-QOL 8-item				
How would you rate your quality of life				nc
How satisfied are you with your health				nc
Do you have enough energy for everyday life	b130			
How satisfied are you with your ability to perform your daily activities		d230		
How satisfied are you with yourself				nc
How satisfied are you with your personal relationships		d750,d760		
Have you enough money to meet your needs				nc
How satisfied are you with the conditions of your living place				nc
24-Hr MQoLQ				
Have increased sensitivity to light or sound			e240,e250	
Have nausea	b535			
Have throbbing head pain	b280			
Feel upset about having migraine headaches	b152			
Feel physically uncomfortable				nd
Feel concerned that your migraine medication wouldn’t relieve your migraine headache symptoms	b152			

Do normal everyday work (job outside the home, schoolwork, housework)		d850,d830,d640, d650,d620		
Stay alert				
Operate machinery or a motor vehicle (including home appliances and office equipment).	b110			
Enjoy life				nc
Interactions with people who are close to you		d750,d760		
Interactions with other people		d710,d720,d730, d740		
Energy level	b130			
Ability to have a good night's sleep	b134			
Mood	b152			
CHQ				
Avoided leaving the house				nd
Avoided making plans due to unpredictability of CH e.g., holidays		d230		
Felt unable to complete duties at work		d850		
Had difficulty in getting involved in leisure activities e.g., cinema, theatre, etc.?		d920		
Avoided crowded and noisy places e.g., public transport, pubs, etc.			e215,e250	
Felt that the severity of cluster headache affected your daily activities		d230		
Been less involved in family affairs e.g., interaction with children, planning holidays		d760		
Been unable to socialise/spend time with friends and family		d750,d760		
Been unable to achieve your daily goals and carry out routines and chores		d230		
Felt less respected by others				nc
Had problems with close personal relationship		d760,d770		
Felt you were a burden on family and friends				nc
Felt self-conscious and uncomfortable about your appearance after a cluster headache attack (e.g., swelling/redness of eyes and facial sweating, etc.)	b152,b144			
Felt that others are dismissive of your cluster headaches				nd
Felt aggressive	b130,b152			
Felt bad about yourself, lost self-confidence or felt worthless	b152			
Felt like harming yourself or suicidal	b152			
Been irritable, impatient or less tolerant	b152			
Been forgetful e.g., missed appointments	b144			
Been unable to take care of your appearance (e.g., take a bath, put make-up on,		d510,d520,d540		

change clothes etc.)				
Felt isolated, lonely or vulnerable	b152			
Found your pain is unbearable if untreated	b160,b280			
Dreaded that the headache would not go away	b160,b280			
Felt lacking in energy and constantly tired	b130			
Felt sleepy, worn out or less able to concentrate due to nocturnal attacks of CH	b134,b140	d160,d161		
Had problems concentrating e.g., reading paper, watching TV, etc.		d160,d161,d166, d110		
Been unable to think clearly	b160			
Felt tense or anxious	b152			
